# A quick and sensitive diagnostic tool for detection of *Maize streak virus*

**DOI:** 10.1038/s41598-020-76612-2

**Published:** 2020-11-12

**Authors:** Mathias Tembo, Adedapo O. Adediji, Sophie Bouvaine, Patrick C. Chikoti, Susan E. Seal, Gonҫalo Silva

**Affiliations:** 1 Zambia Agriculture Research Institute, Mount Makulu Research Station, P/Bag 7, Lusaka, Zambia; 2 Department of Crop Protection and Environmental Biology, Faculty of Agriculture, University of Ibadan, Ibadan, Oyo State Nigeria; 3 Natural Resources Institute, University of Greenwich, Central Avenue, Chatham Maritime, ME4 4TB Kent UK

**Keywords:** Biotechnology, Molecular biology, Plant sciences

## Abstract

Maize streak virus disease (MSVD), caused by *Maize streak virus* (MSV; genus *Mastrevirus*), is one of the most severe and widespread viral diseases that adversely reduces maize yield and threatens food security in Africa. An effective control and management of MSVD requires robust and sensitive diagnostic tests capable of rapid detection of MSV. In this study, a loop-mediated isothermal amplification (LAMP) assay was designed for the specific detection of MSV. This test has shown to be highly specific and reproducible and able to detect MSV in as little as 10 fg/µl of purified genomic DNA obtained from a MSV-infected maize plant, a sensitivity 10^5^ times higher to that obtained with polymerase chain reaction (PCR) in current general use. The high degree of sequence identity between Zambian and other African MSV isolates indicate that this LAMP assay can be used for detecting MSV in maize samples from any region in Africa. Furthermore, this assay can be adopted in minimally equipped laboratories and with potential use in plant clinic laboratories across Africa strengthening diagnostic capacity in countries dealing with MSD.

## Introduction

Maize (*Zea mays* L.) is a major food crop and critical for food security in sub-Saharan Africa (SSA), grown mainly by smallholder farmers on more than 25 million hectares^1^. However, maize yields in SSA range from 1.5 to 2 tonnes per hectare (t ha^–1^) and remain the least compared to other regions worldwide where yields in West Asia and North Africa (6.29 t ha^−1^), East Asia (5.27 t ha^−1^), North America and the developed countries (9.86 t ha^−1^), have been reported^2,3^. In Zambia, maize is grown throughout the country of which about 80% produced by the majority small-holder farmers is estimated at 2,394,907 metric tons cultivated on an area of 1,086,000 ha with average yields of 2.2 t/ha^4^. The country has one of the highest amounts of maize consumed (over 100 kg/capita/year) representing more than 50% of total calories^2^.

A major biotic constraint to maize production in Africa is maize streak virus disease (MSVD) caused by *Maize streak virus* (MSV) (genus *Mastrevirus*, family *Geminiviridae*)^5,6^. Depending on the cultivar grown and the time of infection, yield losses caused by MSV have been reported to range up to 100%^7,8^ translating to US$120 M and US$480 M per year^5^. MSV is the most widely studied mastrevirus^9^ with several strains described (MSV- A, -B, -C, -D, -E, -F, -G, -H, -I, -J and –K), although only MSV-A causes the economically important MSD^10^. The disease is widely distributed across the African continent and in adjacent islands with occurrences of periodic outbreaks that severely devastate maize yields^11^. MSVD is considered as the biggest threat to the food security and economic empowerment in SSA^11^ due to low per-hectare yields in disease ravaged regions.

Within infected maize fields, MSVD symptoms are often difficult to distinguish from those caused by other viruses as observed with Maize stripe virus (MSpV)^12^. Therefore, the use of symptoms for disease diagnosis becomes unreliable since symptoms differ subject on the virus strain, the presence of any mixed viral infections, the cultivar and growth stage, growing environment, and the similarity of viral symptoms to those induced by environmental injury^13^.

Detection methods such as immunosorbent electron microscopy, enzyme-linked immunosorbent assay (ELISA)^14^, Southern blot hybridisation^15^ and polymerase chain reaction–restriction fragment length polymorphism (PCR–RFLP) have been developed to distinguish MSV strains^16^. Although PCR-based tests are sensitive, they require DNA purification, advanced instruments and skilled technicians which make them laborious and time-consuming^17^. In many African countries including Zambia, where there is inadequate laboratory equipment, isothermal amplification systems such as loop-mediated isothermal amplification (LAMP) offer a useful alternative. Lately, numerous LAMP assays have led to the development of several phytopathological diagnostic protocols for the early detection of many diseases caused by viruses, however, none has been developed for the detection of MSV^18^.

LAMP is a technique that uses six primers that recognise eight distinct regions in the target DNA, making it highly specific and with increased amplification efficiency than other DNA amplification methods^19,20^. LAMP assays run at a single temperature of around 65 °C and provide results in less than 30 min due to strand displacing DNA polymerases that display faster reaction kinetics^21^. Additionally, the LAMP chemistry exhibits tolerance of substances which are inhibitory to PCR, allowing versatility for on-site diagnostics of plant pathogens^21,22^.

Isothermal technologies are increasingly being developed particularly for low-resource regions where infrastructure, equipment, and skills to support the use of PCR as a diagnostic tool are lacking^23^. The current availability of several portable fluorescence-reading LAMP devices such as the Genie II (Optigene Ltd., West Sussex, UK) which offsets the need of a high-cost equipment used for thermal cycling, mean that this assay could be a promising alternative to conventional PCR in current general use and could increase diagnostic capacity in different maize-growing regions in Africa. In this study, the development and application of LAMP as a quick method for MSV detection are described.

## Results

### Specificity and sensitivity of LAMP method

**Figure 1:**
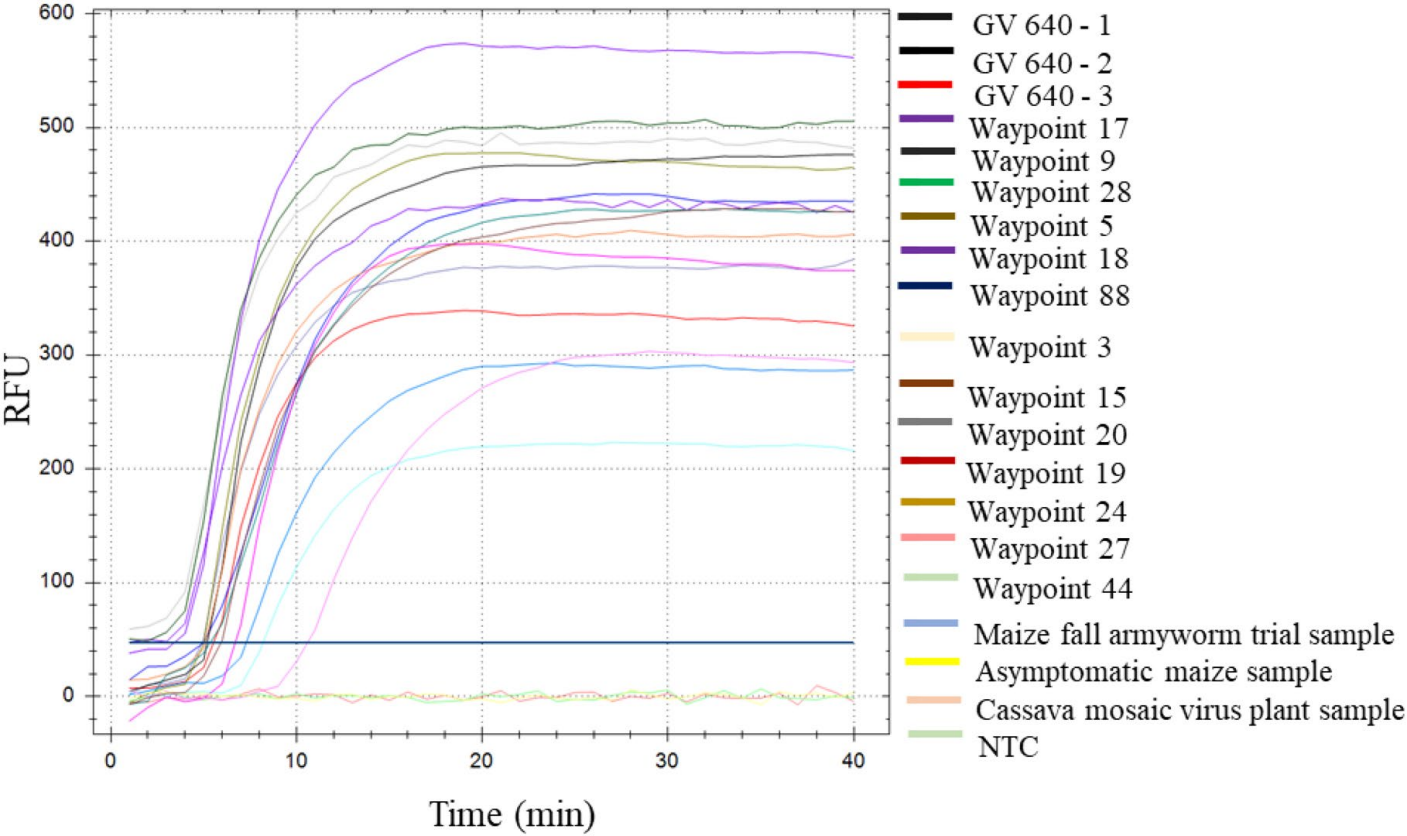
Amplification plot of real-time LAMP for MSV detection. Normalised fluorescence intensities were plotted against time in minutes. The solid bar corresponds to the threshold line (graph generated by Bio-Rad CFX Manager v3.1 software). NTC refers to the non-template control (water control). Cassava sample infected with cassava mosaic begomovirus was used to test specificity of LAMP assay. Sample details are given in Table 1.

**Figure 2:**
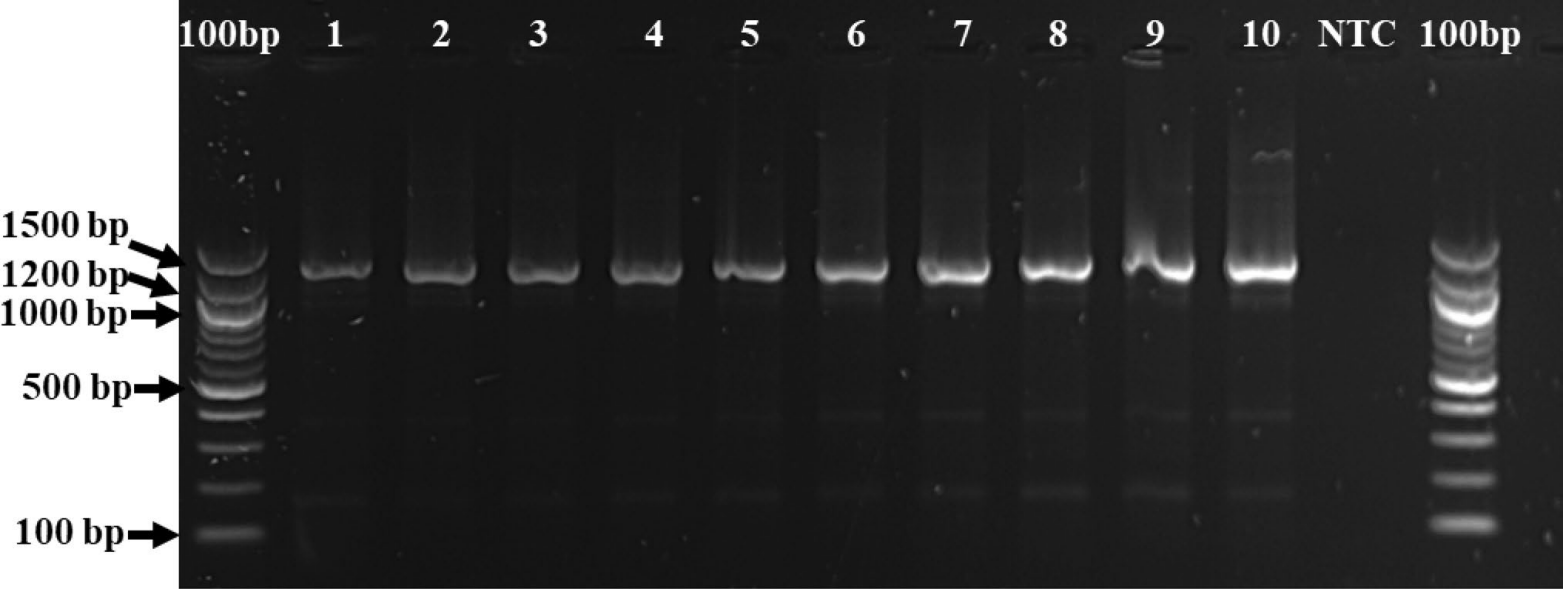
Gel electrophoresis of the amplified PCR products of MSV- positive maize samples. The PCR results of all samples tested can be found in Supplementary Figure S5 online.

To evaluate the suitability of LAMP for the detection of MSV, DNAs obtained from 26 symptomatic maize plants were screened by both the new LAMP assay and the PCR method in current general use. The LAMP assay was performed in a real-time thermal cycler set at 65 °C for 40 min (Fig. 1). All samples tested positive for MSV with results typically obtained within 10 min, suggesting a high virus titre in the samples tested. The same DNAs were analysed by PCR (Fig. 2) using primers designed by Martin et al^10^. All 26 samples tested positive for MSV by PCR (Table 1).

**Table 1:** List of sampled locations with coordinates for MSV-LAMP and PCR results, Zambia, 2019 (+ = MSV positive; −= MSV negative).

Sample name	District	Latitude	Longitude	Altitude	LAMP result	PCR result
Wp 01	Chembe	11.776195	28.745825	1227.6	+	+
Wp 03	Chembe	11.649354	28.731392	1356.7	+	+
Wp 05	Mansa	11.36456	28.83456	1293.4	+	+
Wp 09	Mansa	11.01725	28.931224	1313.5	+	+
Wp 13	Chipili	10.49756	29.212317	1323	+	+
Wp 15	Chipili	10.340273	29.449346	1291.25	+	+
Wp 18	Luwingu	10.226266	29.887695	1426.98	+	+
Wp 19	Mansa	11.115	28.881412	1249.4	+	+
Wp 20	Mansa	11.03533	28.841111	1258.14	+	+
Wp 24	Mwense	10.389962	28.664133	924.46	+	+
Wp 25	Mwansabombwe	9.982072	28.717581	948.91	+	+
Wp 27	Chienge	9.133285	28.881494	991.1	+	+
Wp 28	Chienge	9.100374	28.955965	965.95	+	+
Wp 36	Chienge	9.075585	29.30655	955	+	+
Wp 40	Kaputa	9.156244	29.300292	1004.9	+	+
Wp 44	Mporokoso	9.470472	29.629855	1322.48	+	+
Wp 49	Mporokoso	9.402938	30.088255	1398.14	+	+
Wp 54	Mporokoso	9.473235	30.518845	1410.08	+	+
Wp 58	Lunte	9.785358	30.750457	1534.37	+	+
Wp 74	Kasama	10.547187	31.192464	1233.62	+	+
Wp 82	Chinsali	10.656782	31.924028	1327	+	+
Wp 84	Chinsali	10.663401	32.125274	1372.41	+	+
Wp 88	Serenje	13.074607	30.562672	1612.97	+	+
GV 640-1	Lusaka	15.547648	28.249297	1226.55	+	+
GV 640-2	Lusaka	15.547648	28.249297	1226.55	+	+
GV 640-3	Lusaka	15.547648	28.249297	1226.55	+	+
Maize fall armyworm trial	Lusaka	15.547648	28.249297	1226.55	+	+
Asymptomatic maize sample	Mount Makulu	15.547648	28.249297	1226.55	−	−
Cassava sample	Mount Makulu	15.547648	28.249297	1226.55	−	−

**Figure 3:**
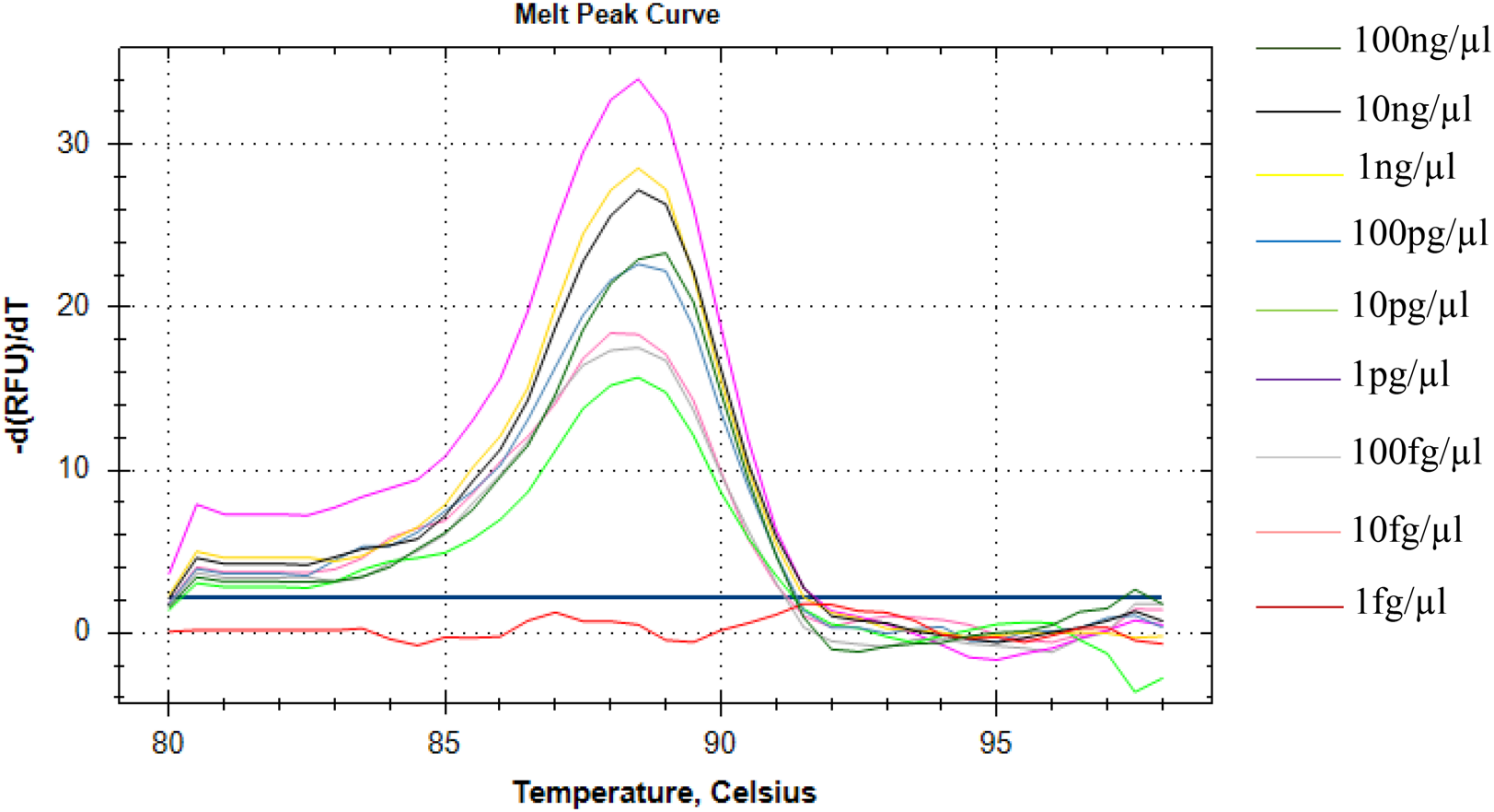
Melt curve profiles of MSV LAMP amplicons. A melting temperature of 88.5 °C was observed for all amplification products (graph generated by Bio-Rad CFX Manager v3.1 software).

The specificity of the LAMP assay was tested using DNAs obtained from asymptomatic maize plants as well as cassava plants infected with geminiviruses (cassava mosaic begomoviruses, CMBs). No cross reactions occurred for these samples, (Table 1; Fig. 1) and LAMP primers produced a single peak melt curve (Fig. 3) confirming the specificity of the amplified product. No melt peaks were detected for the negative controls.

**Figure 4:**
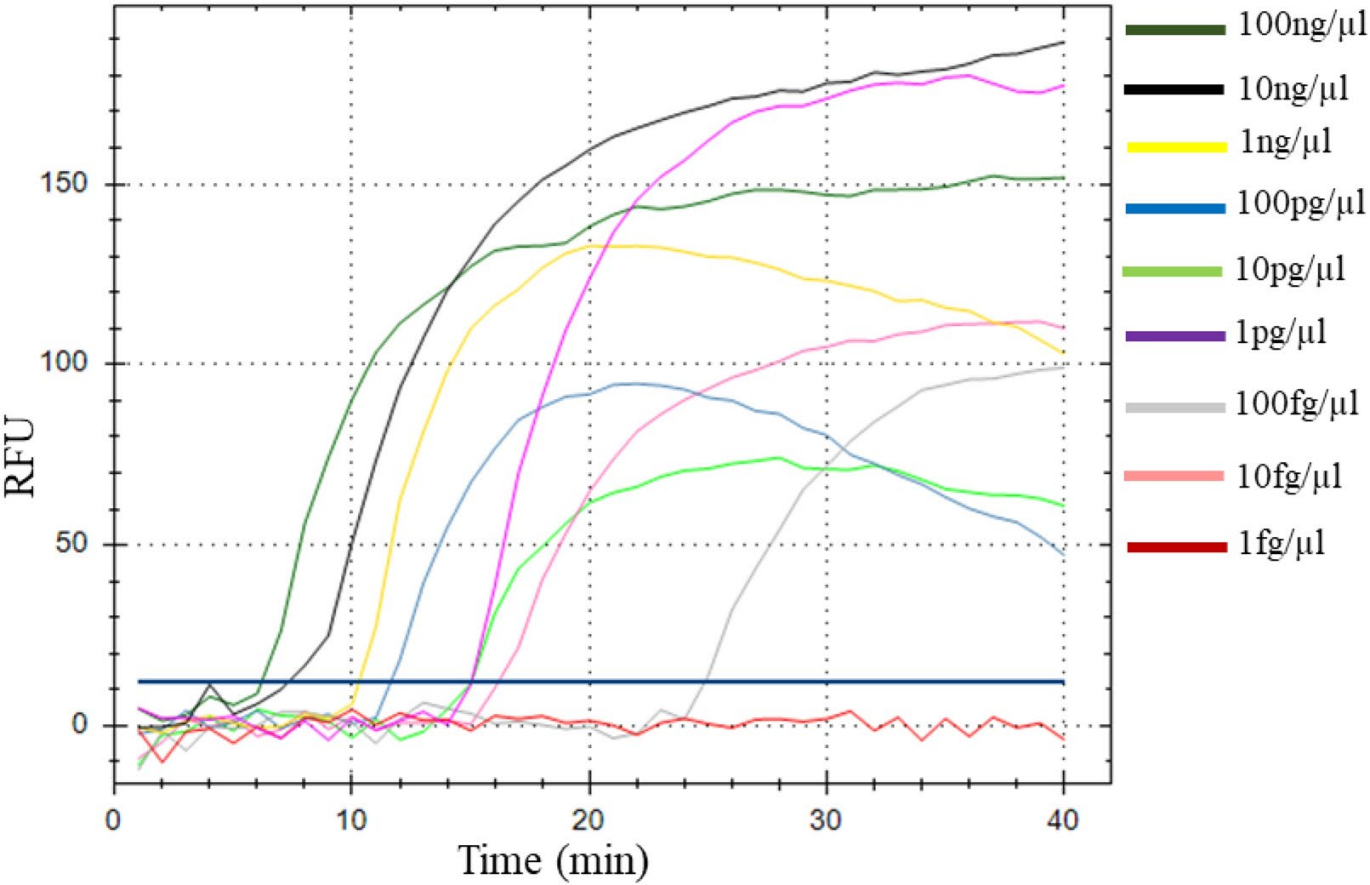
Sensitivity of the real-time LAMP assay for MSV detection. Normalised fluorescence intensities were plotted against time in minutes. The solid bar corresponds to the threshold line (graph generated by Bio-Rad CFX Manager v3.1 software).

**Figure 5:**
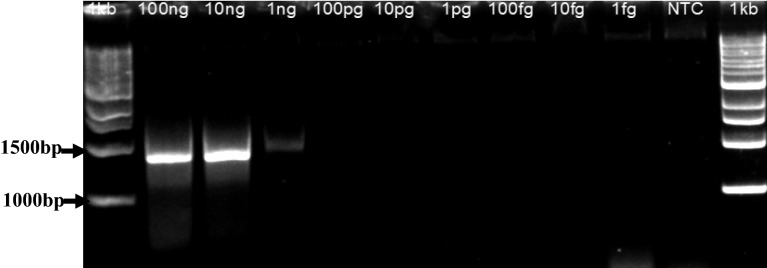
Sensitivity of conventional PCR for the detection of MSV.

To determine the detection limit of the LAMP assay, reactions were prepared with tenfold serially diluted DNA extracts obtained from a MSV-infected maize plant and by comparing results with PCR. Each dilution was tested in three independent assays. A representative assay is shown in Fig. 4. For the LAMP assay, positive results were consistently observed for samples containing DNA concentrations ranging from 100 ng/μl up to 10 fg/μl. None of the samples with a DNA concentration of 1 fg/μl were detected as positive (Fig. 4). The time required to detect MSV in the most diluted sample (i.e. containing a DNA concentration of 10 fg/μl) was around 25 min, suggesting that 40 min for data collection period is more than sufficient to detect MSV-positive samples. In contrast, the detection limit for the conventional PCR was 1 ng/μl (Fig. 5) indicating that the LAMP assay developed in this study is at least 10^5^ times more sensitive than PCR.

### Sequence identities of MSV amplicons

PCR products of MSV-positive samples were sequenced and used for phylogenetic and diversity analysis. The mean pairwise nucleotide diversity between the MSV sequences obtained in this study was 97.6%. Nucleotide pairwise comparison of these sequences with MSV sequences deposited in GenBank revealed 93.9–99.7% nucleotide identities (see Supplementary Data S1 online).

**Figure 6:**
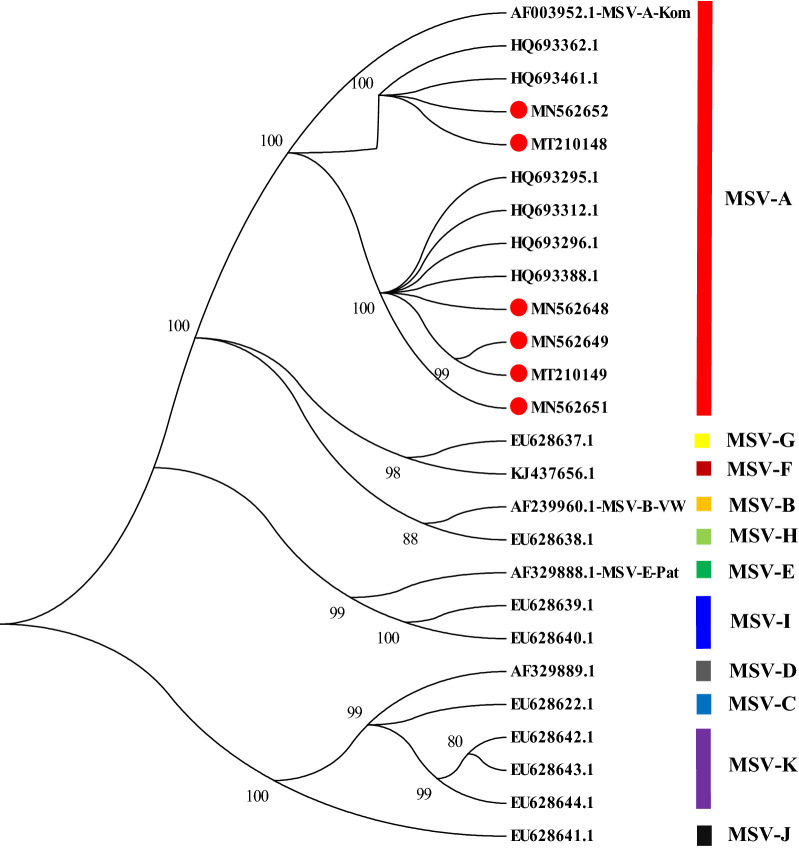
Phylogenetic tree based on 1300-bp partial nucleotide sequences spanning the RepA and the 5′ of MP region of MSV sequences obtained in this study (*Maize streak virus* MSV-A_ZMB_Chip_13 (MN562648), *Maize streak virus* MSV-A_ZMB_Mwan_25 (MN562649), *Maize streak virus* MSV-A_ZMB_Mpor_54 (MN562651), *Maize streak virus* MSV-A_ZMB_Kasa_74 (MN562652), *Maize streak virus* MSV-A_ZMB_Chie_36 (MT210148) and *Maize streak virus* MSV-A_ZMB_Chin_84 (MT210149)) and reference MSV sequences (*Maize streak virus* – A_Kom (AF003952.1), *Maize streak virus* – B_Vaalhart wheat (AF239960.1), *Maize streak virus*—E[Pat] isolate MSV-Pat (AF329888.1), *Maize streak virus* – D_Raw (AF329889.1), *Maize streak virus* MSV-C_Ug-LuwA-2007 (EU628622.1), *Maize streak virus* MSV-G_Pas-Jic16-2002 (EU628637.1), *Maize streak virus* MSV-H_Ng-Lag-g74-2007 (EU628638.1), *Maize streak virus* MSV-I_Za-NewA-g217-2007 (EU628639.1), *Maize streak virus* MSV-I_Za-RosC-g130-2006 (EU628640.1), *Maize streak virus* MSV-J_Zw-Mic24-1987 (EU628641.1), *Maize streak virus* MSV-K_Ksv-Jic2-2002 (EU628642.1), *Maize streak virus* MSV-K_Ug-BusD-2005 (EU628643.1), *Maize streak virus* MSV-K_Zw-Mic23-1987 (EU628644.1), *Maize streak virus* MSV-A_CF_Bang1_Car39-2008 (HQ693295.1), *Maize streak virus* MSV-A_CF_Bang10_Car49-2008 (HQ693296.1), *Maize streak virus* MSV-A_CF_Bos6_Car6-2008 (HQ693312.1), *Maize streak virus* MSV-A_MZ_Pem1_Moz36-2007 (HQ693362.1), *Maize streak virus* MSV-A_NG_Ogb1_N36a-2007 (HQ693388.1), *Maize streak virus* MSV-A_ZM_Kab1_Z24-2008 (HQ693461.1) and *Maize streak virus* MSV_F_NG_ng17_Sam_2011 (KJ437656.1) obtained from GenBank. Bootstrap analysis conducted in MEGA 7 was performed with 1000 replicates and branches corresponding to partitions reproduced in less than 80% bootstrap replicates are collapsed. The percentage of replicate trees in which the associated taxa clustered together in the bootstrap test (1000 replicates) are shown next to the branches. MSV sequences obtained in this study and indicated with red solid dots were processed using Chromas v2.6.6 software (https://technelysium.com.au/wp/chromas/).

Phylogenetic and percentage identity analyses confirmed that the MSV Zambian sequences from this study (*Maize streak virus* MSV-A_ZMB_Chip_13 (MN562648), *Maize streak virus* MSV-A_ZMB_Mwan_25 (MN562649), *Maize streak virus* MSV-A_ZMB_Mpor_54 (MN562651), *Maize streak virus* MSV-A_ZMB_Kasa_74 (MN562652), *Maize streak virus* MSV-A_ZMB_Chie_36 (MT210148) and *Maize streak virus* MSV-A_ZMB_Chin_84 (MT210149)) clustered with others isolated from the MSV-A strain (Fig. 6). The clustering of MSV Zambian sequences did not correlate with their country of origin. The Zambian MSV sequences obtained here are closely related to MSV sequences obtained from different countries in Africa. MSV-A_ZMB_Chie_36 (MT210148) had the highest nucleotide identity (99.7%) with MSV accessions HQ693362 and HQ693461 from Mozambique and Zambia, respectively whilst the lowest nucleotide sequence identity was obtained for MSV-A_ZMB_Mpor_54 (MN562651) which had 93.9% identity to an MSV accession from Cameroon (HQ693327) (see Supplementary Sata S1 online).

## Discussion

Maize streak virus disease (MSVD) is caused by *Maize streak virus* (MSV), a geminivirus that occurs in most sub-Saharan countries. MSVD is regarded as the third most devastating disease of maize worldwide after northern corn leaf blight (NCLB) and gray leaf spot (GLS)^24^. In Africa, however, where maize is a staple food, MSVD is the most important disease affecting maize crops and poses a big threat to food security in the region^5^. The MSV can cause up to 100% yield losses^25^ and therefore has potential to cause a devastating effect not only on the maize crop and the livelihoods of the resource-poor farmers in Zambia, but also on other key actors in the maize seed/grain value chain, especially seed companies and processors with consequent losses of sales. The development of robust, quick and efficient methods for the precisely diagnosis of MSVD is vital towards appropriate disease control and for increased food and income security.

In this study, a LAMP method was developed for the detection of MSV and compared to the standard PCR method for MSV diagnosis. The LAMP assay was 10^5^ times more sensitive in detecting MSV than standard PCR and with high specificity as no cross-reactions with closely related geminiviruses were detected.

The detection of LAMP products was achieved using a laboratory real-time PCR instrument to measure fluorescence. Alternative detection methods such as agarose gel electrophoresis can be used^26^ but these increase the risk of contamination through the opening of reaction tubes containing LAMP-amplified products^18^. LAMP reactions generate five to ten more amplicons than a standard PCR^27,28^. To avoid carry-over contamination issues it is very important not to open the tubes after a LAMP reaction. Although the real-time detection used in our study avoided any risk of contamination associated with post-amplification processes there are several low-cost portable fluorescence-reading instruments (e.g. Genie II (Optigene Ltd., West Sussex, UK), Bioranger (Diagenetix, Inc., Honolulu, HI, USA) and Genelyzer FIII (Canon Medical Systems, Tochigi, Japan)) available in the market that can be used to measure the fluorescence signal and thereby reducing the cost of the assay^18,30,31^. These simple battery-powered instruments offer a major advantage of the LAMP assay in places experiencing repeated power failures as reactions could proceed even without electricity.

The developed LAMP assay has more advantages compared to the standard PCR currently in general use for MSV diagnosis. In this study the reaction time of the LAMP assay was less than 40 min compared to > 90 min required for the PCR plus the time required for gel electrophoresis. Previous reports have described the use of LAMP in conjunction with crude plant extracts avoiding total DNA extractions^19,32^, reducing costs^18^, shortening the processing time and facilitating the use of the method in low-resource settings^32^. In contrast to LAMP, PCR needs more stringent conditions such as expensive equipment to perform thermal cycling steps at higher temperatures and highly specialised personnel. For these reasons, the LAMP developed here appears to be a promising alternative to PCR for testing for MSV.

Due to budget restrictions, only a small number of MSV sequences were obtained in this study. Nevertheless, the high degree of sequence identity between the Zambian and other African MSV isolates suggest that this LAMP assay can be used for detecting MSV in maize samples from any region in Africa.

The developed LAMP will contribute not only to improve the diagnosis of MSV but also to strengthen the molecular diagnostic testing capability at the Zambia Agriculture Research Institute. It is paramount that the institute is equipped with appropriate diagnostic methods to detect the most important viruses present in maize to allow the development and application of appropriate control measures in maize cultivations in Zambia.

Future studies will investigate procedures to adapt the LAMP assay as a surveillance and early-warning tool for the presence of MSV and/or other pathogens as well as detection of MSV in leafhoppers to study the combined leafhopper/MSV diversity present in Zambian maize fields. In the short term, however, the LAMP assay developed in this study could be immediately employed for sanitary selection and in eradication programmes^18^.

## Conclusion

In this study a specific and sensitive LAMP assay for the detection of MSV was developed. This assay was able to detect MSV from field maize plants and was 10^5^ times more sensitive than conventional PCR currently in general use. The LAMP assay has the potential to be adopted for routine detection of MSV in maize breeding programs and/or certification laboratories. Most importantly, the developed method has the immediate impact of strengthening the molecular diagnostic testing capability at the Zambia Agriculture Research Institute.

## Materials and methods

### Plant material and DNA extraction

Maize leaf samples (n = 26) were obtained from MSV symptomatic maize plants from 13 districts (Chembe, Mansa, Chipili, Luwingu, Mwense, Mwansabombwe, Chienge, Kaputa, Mporokoso, Lunte, Kasama, Chinsali and Serenje) in Zambia. The districts are in the agro-ecological region III of Zambia which experiences the highest rainfall of 1000 mm and above per annum^33^ with the potential for high maize production and at the same time regarded as a hotspot for MSV. Genomic DNA for the maize and cassava (used in the determination of LAMP specificity) leaf samples was extracted using a modified cetyltrimethylammonium bromide (CTAB) procedure as described by Lodhi et al.^34^. The DNA concentration and quality were determined using NanoDrop 2000 (ThermoFisher Scientific, UK). The detailed DNA concentrations and dilutions for the different samples can be found as Supplementary Data S2 and S3 online, respectively.

### PCR amplification of MSV sequences

Screening of samples for the presence of MSV was performed by PCR using the degenerate primer set (5′-TTGGVCCGMVGATGTASAG-3′; 5′-CAAAKDTCAGCTCCTCCG-3′), amplifying a 1300 bp region spanning the Replication associated (RepA) and the 5′ half of the Movement protein (MP) genes of the MSV genome^10^. PCR amplifications were set up in 25 µL reactions containing 10 ng of DNA template, 10 µM of each primer, 10 mM of dNTPs mix, 2.5 U/µL DreamTaq DNA Polymerase and 10 × DreamTaq Green buffer (Thermo Scientific, Loughborough, UK) containing 25 mM MgCl_2_. The cycle conditions for PCR amplification were 94 °C for 1 min, followed by 30 cycles of 93 °C for 45 s, 54 °C for 30 s, 72 °C for 1.5 min and a final extension of 72 °C for 10 min^16^. PCR products were analysed by agarose gel electrophoresis, purified using the GeneJET PCR Purification Kit (Fermentas, UK) following manufacturer’s instructions and Sanger sequenced by the Source BioScience sequencing service (Cambridge, UK).

### Phylogenetic and sequencing analysis

The sequenced MSVs were submitted to GenBank using Bankit, a web-based data submission tool^35^. The quality of the virus sequences was processed by removing any low quality sequence using Chromas Software Version 2.6.6 (https://technelysium.com.au/wp/chromas/)^36^. Quality scores of 0.05 were used for trimming and sequences with scores below 50% were excluded from analyses. The single contiguous sequences (contigs) were generated from the two separate sequence files using the CAP3 sequence assembly program^37^. The sequences were analyzed against sequences available in GenBank using the basic local alignment search tool (BLASTn)^38^ at the NCBI website (https://www.ncbi.nlm.nih.gov/genbank/). The MSV sequences from the database that had the highest similarity to each BLAST query sequence were selected for subsequent sequence similarity and phylogenetic analysis. Nucleotide sequences were aligned using the MAFFT v7.450 alignment program^39^ with default option settings. The evolutionary history was inferred using the Neighbor-Joining method^40^. The evolutionary distances were computed using the Jukes-Cantor method^41^ and in the units of the number of base substitutions per site. Evolutionary analyses were conducted in MEGA7^42^. The analyses included the MSV Zambian sequences obtained in this study (GenBank accession numbers MN562648, MN562649, MN562651, MN562652, MT210148 and MT210149) and published MSV full genome sequences comprising of 192 MSV isolates described^6^. The robustness of each tree was determined by generating a bootstrap consensus tree using 1000 replicates. Virus sequences obtained from GenBank were used for comparative analyses and accession numbers shown in the phylogenetic tree. Pairwise identity comparisons of nucleotide sequences were performed using the multiple alignment tool of the software GENEIOUS v11.1.5^43^.

### LAMP primer design

LAMP primers (Table 2) were designed to amplify a fragment between the MP and the coat protein (CP) genes of MSV. This was done through a multiple sequence alignment of complete MSV nucleotide sequences available in GenBank. Primers were designed using PrimerExplorer v5 software (available at https://primerexplorer.jp/e/) with default settings. The targeted region of the LAMP primers has 70 bp overlap with the region covered by the PCR primers used in our study. Degenerate bases were incorporated into the LAMP primers to mitigate against any intraspecific variation between MSV isolates. BLAST analysis indicated that primers’ regions selected for assay had high homology between MSV isolates and showed no putative cross-reaction with other members of the family *Geminiviridae*.

**Table 2:** LAMP primers designed in this study for the detection of *Maize streak virus* (MSV) using the Genbank accession AF003952 as reference (primers were designed using PrimerExplorer v5 software (available at https://primerexplorer.jp/e/)).

Primer name	Sequences (5′ → 3′)	Position of primer	Final concentration (μM)
MSV_F3	TGGGTGCTGAGAGAYCTTA	294–312	0.2
MSV_B3	TCATCKCCMCGCTTCCTC	500–483	0.2
MSV_FIP	AGGGTTGCTCCTATCCACAGCT-TGAAGGCTCGACAAGGCA	392–371	0.8
MSV_BIP	ACCAAGTCAGGGCAATCCCG-TTGGACGTGGACATGGCT	413–432	0.8
MSV_LoopF	ATCAGCTCCTCCGTGGATC	358–340	0.4
MSV_LoopB	GCCATTTGTTCCAGGCACG	434–452	0.4

### LAMP assay reaction, sensitivity and specificity

The extracted maize DNA were used as templates in LAMP reactions performed with the C1000 Touch Thermocycler (BioRad, USA) following the protocol described by Panek and Frąc^44^. The LAMP reactions were performed in 25 µl total volumes containing 1 × Isothermal MasterMix (ISO-001, OptiGene), 0.2 µM each external primer (F3/B3), 0.8 µM each internal primer (FIP/BIP), 0.4 µM each loop primer (loop-F/loop-B) and 10 ng of maize DNA (see Supplementary Data S2). Reactions containing water instead of DNA were included in each run as non-template controls (NTC). LAMP reactions were incubated at 65 °C for 40 min with fluorescence measurements in the FAM channel obtained every minute^44^. Fluorescence data was normalized and baseline adjusted using Bio-Rad CFX Manager v3.1 software. The obtaining results were visualized as amplification plots (ΔR) expressed as relative fluorescence units (RFU) in time^44^.

Cassava plants infected with CMBs (*African cassava mosaic virus* and *East African cassava mosaic virus*) (family *Geminiviridae*) were obtained from the plant pathology screen house at Mount Makulu Research Station, Zambia Agriculture Research Institute (ZARI) and included in the LAMP reaction to test the specificity of the LAMP assay. A multiplex PCR^45,46^ was used to confirm the presence of CMBs in the cassava plants (Supplementary Fig. S4 online).

To determine the detection limit of the LAMP assay and the PCR, serial dilutions of DNA obtained from MSV-infected plant was prepared. Dilutions were made with DNase-free water in a tenfold series with concentrations of 100 ng/μl, 10 ng/μl, 1 ng/μl, 100 pg/μl, 10 pg/μl, 1 pg/μl, 100 fg/μl, 10 fg/μl and 1 fg/μl of which 1 μl each was used as template for LAMP and PCR^44^. All reactions were performed in triplicates. LAMP reactions were incubated with fluorescence measurements as described above following the procedures described by Panek and Frąc^44^. To measure the melting temperature of the amplification products, the reactions were subjected to a slow annealing step from 65 to 95 °C (with 0.05 °C/s increments) with fluorescence monitoring^44^. The obtaining results were visualized as amplification plots (ΔR) expressed as the negative value of the change in RFU over the change in temperature (− dRFU/dT) versus temperature (degrees Celsius).

## Supplementary information

Supplementary Data 1.

Supplementary Data 2.

Supplementary Data 3.

Supplementary Figure S4.

Supplementary Figure S5.
