# Supplementary material for: A quick and sensitive diagnostic tool for detection of Maize streak virus
Source: Sci Rep. 2020 Nov 12;10:19633. doi: 10.1038/s41598-020-76612-2 (PMC7661706; doi:10.1038/s41598-020-76612-2)
Supplement: Supplementary file 4 — Supplementary Figure S4. [file 41598_2020_76612_MOESM4_ESM.docx]

**A quick and sensitive diagnostic tool for detection of Maize streak virus**

Mathias Tembo^1^*, Adedapo O. Adediji^2^, Sophie Bouvaine^3^, Patrick C. Chikoti^1^, Susan E. Seal^3^ & Gonҫalo Silva^3^

^1^Zambia Agriculture Research Institute, Mount Makulu Research Station, P/Bag 7, Lusaka, Zambia.
^2^Department of Crop Protection and Environmental Biology, Faculty of Agriculture, University of Ibadan, Ibadan, Oyo State, Nigeria.
^3^Natural Resources Institute, University of Greenwich, Central Avenue, Chatham Maritime, Kent, ME4 4TB, UK.
*Corresponding author: mathiastembo2002@yahoo.com; Tel: +260966957408; Fax: +260211278130.

**368 bp**

**650 bp**

**650 bp**

**368 bp**

**Supplementary figure S4:** Gel electrophoresis of multiplex PCR amplified DNA fragments of cassava plants infected with African cassava mosaic virus (368 bp) and East African cassava mosaic virus (650 bp) using the primers CMBrep/F, ACMVrep/R and EACMVrep/R with 100 bp ladder.
