# Supplementary material for: A quick and sensitive diagnostic tool for detection of Maize streak virus
Source: Sci Rep. 2020 Nov 12;10:19633. doi: 10.1038/s41598-020-76612-2 (PMC7661706; doi:10.1038/s41598-020-76612-2)
Supplement: Supplementary file 5 — Supplementary Figure S5. [file 41598_2020_76612_MOESM5_ESM.docx]

**A quick and sensitive diagnostic tool for detection of Maize streak virus**

Mathias Tembo^1^*, Adedapo O. Adediji^2^, Sophie Bouvaine^3^, Patrick C. Chikoti^1^, Susan E. Seal^3^ & Gonҫalo Silva^3^

^1^Zambia Agriculture Research Institute, Mount Makulu Research Station, P/Bag 7, Lusaka, Zambia.
^2^Department of Crop Protection and Environmental Biology, Faculty of Agriculture, University of Ibadan, Ibadan, Oyo State, Nigeria.
^3^Natural Resources Institute, University of Greenwich, Central Avenue, Chatham Maritime, Kent, ME4 4TB, UK.
*Corresponding author: mathiastembo2002@yahoo.com; Tel: +260966957408; Fax: +260211278130.

**1000 bp**

**1000 bp**

**1500 bp**

**1500 bp**

**1kb 11 12 C1 13 14 15 16 17 18 19 20 21 22 23**

**1kb 24 25 26 C2 C3 NTC 1kb**

**Supplementary Figure S5.** Gel electrophoresis of amplified PCR fragments (1,300 bp) of MSV-positive maize and cassava control samples.
